# Supplementary material for: Volume expansion and TRPV4 activation regulate stem cell fate in three-dimensional microenvironments
Source: Nat Commun. 2019 Jan 31;10:529. doi: 10.1038/s41467-019-08465-x (PMC6355972; doi:10.1038/s41467-019-08465-x)
Supplement: Supplementary file 1 — Supplementary Information [file 41467_2019_8465_MOESM1_ESM.pdf]

Volume expansion and TRPV4 activation regulate stem cell fate in three-dimensional microenvironments

Hong-pyo Lee*, Ryan Stowers*, and Ovijit Chaudhuri

Department of Mechanical Engineering, Stanford University, Stanford, CA 94305, USA

*These authors contributed equally: Hong-pyo Lee, Ryan Stowers

Correspondence and requests for materials should be addressed to O.C. (email: chaudhuri@stanford.edu)

Supplementary Information

Supplementary Figures and Legends

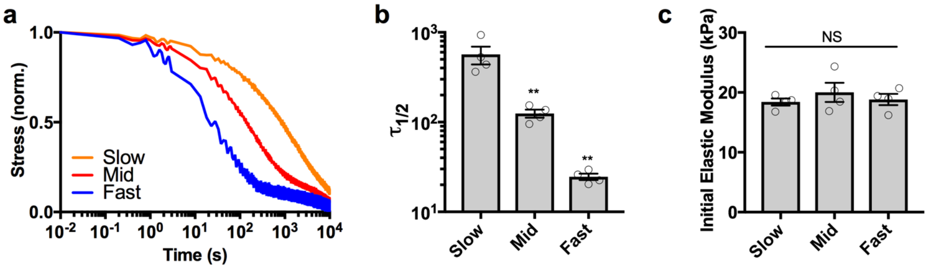

Supplementary Figure 1 | Mechanical properties of alginate hydrogels used for 3D culture. **a**, Representative profiles of stress relaxation tests of hydrogels composed of alginate of varying molecular weights. **b**, Quantification of the time constant indicating rate of stress relaxation for the different alginate hydrogels. **c**, Quantification of the initial modulus of the different alginate hydrogels (n=4 hydrogels for each condition, ** $p<0.01$, and NS $p>0.05$ compared to slow relaxing hydrogels by one-way ANOVA test). All data are shown as mean ± s.e.m.

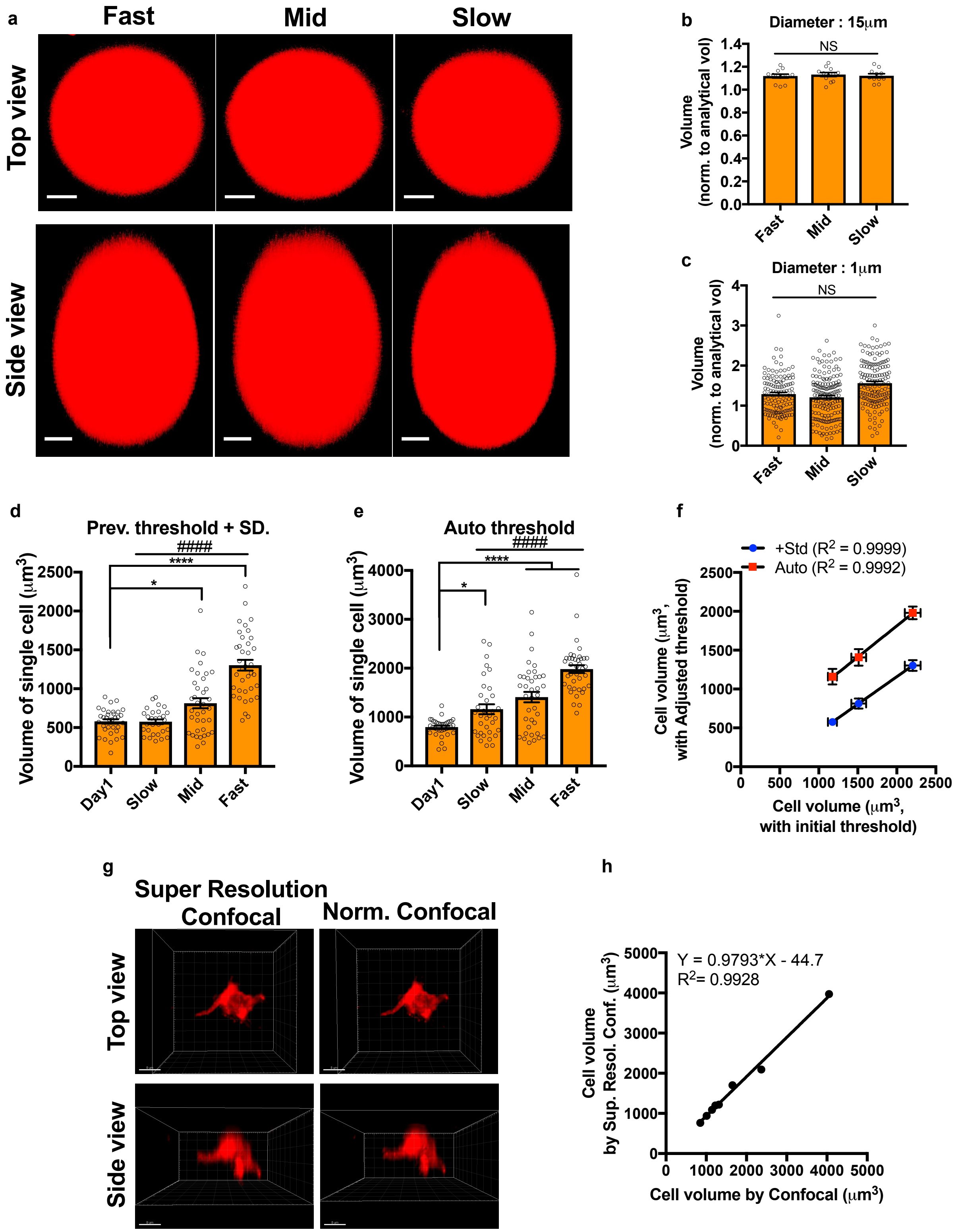

Supplementary Figure 2 | Validation for accuracy of 3D volume measurement. a-b,

Quantification of cell volume with an adjusted threshold increased by the standard deviation of

intensity of membrane dye in each 3D-image (a), and the optimized threshold that Imaris program automatically determines in each 3D-image (b) ($n \geq 35$ single cells from 3 biological replications per each condition, **** $p<0.0001$ and * $p<0.05$ compared to control by one-way ANOVA test, #### $p<0.0001$ by Spearman's rank correlation). **c,** Scatter plots of the cell volume measured with initial threshold or adjusted thresholds. Linear regression analysis indicated each linear correlation between two groups of cell volume (R square = 0.9999 with initial threshold + std and R square = 0.9992 with auto threshold by Imaris). **d,** Top view and side view of representative images of fluorescent microbeads, scale bar = 4 μm. **e-f,** Quantification of the volume of microbeads of 1 μm (e) or 15 μm (f) nominal diameter encapsulated in hydrogels with different rates of stress relaxation. ($n \geq 10$, 15 μm-microbeads and $n \geq 120$, 1 μm-microbeads from 3 replications per each condition, NS $p>0.5$ by one-way ANOVA test). **g,** Top view and side view of representative images with 3D stack of a single cell imaged with normal confocal and super resolution confocal microscopy, scale bar = 8 μm. **h,** Linear regression analysis indicated a linear correlation between two groups of cell volume imaged with normal confocal and super resolution confocal (R square = 0.9928, n = 8 single cells cultured in fast relaxing hydrogel with or without osmotic pressure). All data are shown as mean ± s.e.m.

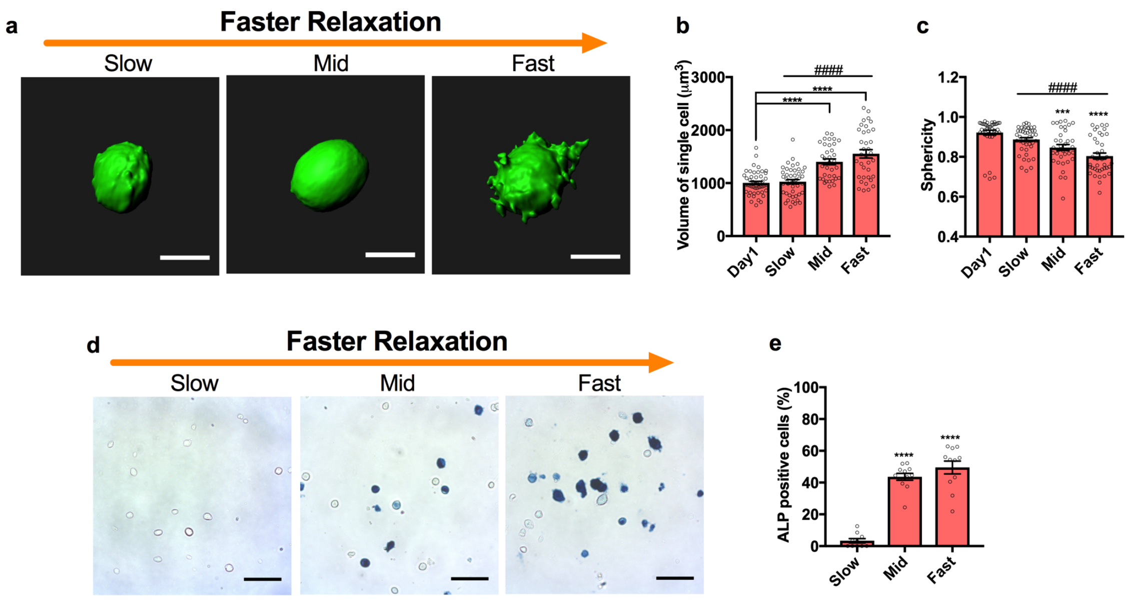

Supplementary Figure 3 | Volume expansion and osteogenic differentiation of MSCs cultured in hydrogels with low RGD density (150 μM) for 7 days. a, Representative 3D images of MSCs cultured in hydrogels with varied levels in stress relaxation after 7 days. Scale bar, 10 μm **b-c**, Quantification of single cells volume (**b**) and sphericity (**c**) (n ≥ 35 single cells from 3 biological replicates per each condition, #### p<0.0001 by Spearman's rank correlation, **** p<0.0001, and *** p<0.001 compared to MSCs cultured in hydrogels for 1 day by one-way ANOVA test). **d**, Representative images of ALP staining for MSCs cultured in hydrogels with varying stress relaxation. Scale bar, 25 μm. **e**, Quantification of the percentage of ALP-positive cells (n ≥ 10 images from 3 biological replications per each condition, **** p<0.0001 compared to slow relaxing hydrogel condition by one-way ANOVA test). All data are shown as mean ± s.e.m.

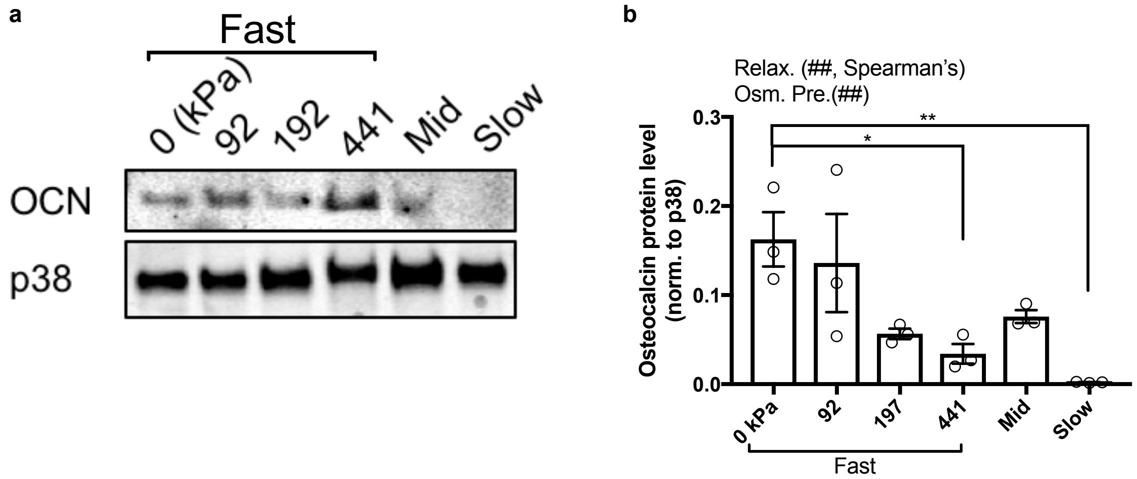

Supplementary Figure 4 | Osteocalcin (OCN) levels of MSCs in hydrogels with varying levels of stress relaxation or hyper-osmotic pressure. a-b, Western blot analysis (a) and quantification (b) of osteocalcin protein levels in MSCs cultured in hydrogel with varying stress relaxation and in fast relaxing hydrogel with altered osmotic pressure for 7 days (** $p<0.01$, and * $p<0.05$ respectively compared to fast stress relaxation and 0 kPa osmotic pressure case by one-way ANOVA test, ## $p<0.01$ by Spearman's rank correlation).

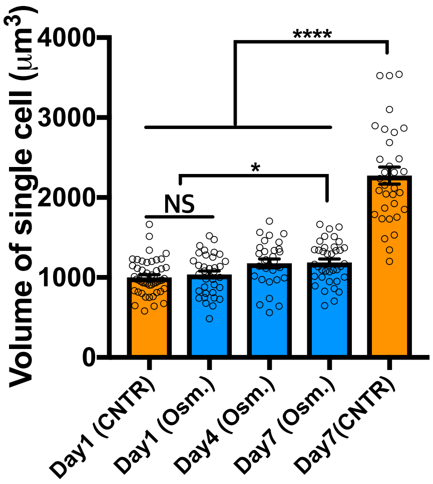

Supplementary Figure 5 | Cell volume of MSCs is stable under increased osmotic pressure. Quantification of volume of single cells cultured in fast relaxing hydrogel with or without osmotic pressure of 197 kPa ($n \geq 35$ single cells from 3 biological replications per each condition, **** $p<0.0001$, * $p<0.05$ and NS $p>0.05$ by one-way ANOVA test). Data are shown as mean ± s.e.m.

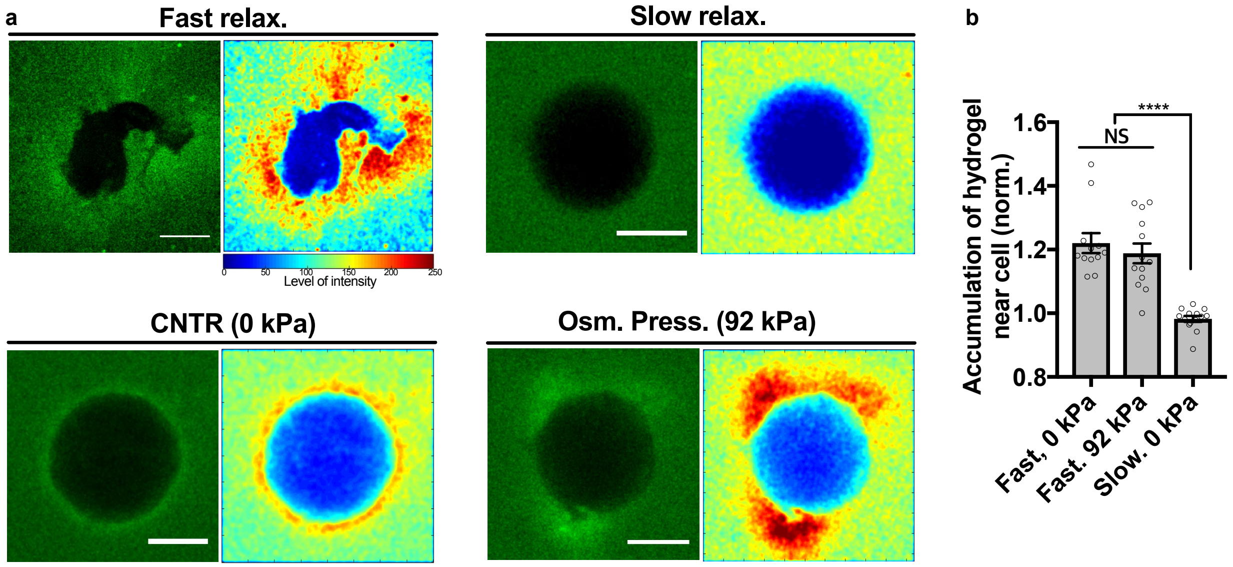

Supplementary Figure 6 | MSCs accumulate alginate on cell periphery in fast relaxing gels, despite hyper-osmotic pressure. a, Representative images and intensity profiles of fluorescent alginate culturing MSCs over 7 days in the indicated conditions. **b,** Quantification of the accumulation of fluorescent alginate by cells cultured in the indicated conditions (n ≥ 10 images from 3 replications per each condition, **** p<0.0001 and NS p>0.5 by one-way ANOVA). Scale bar, 10 µm. All data are shown as mean ± s.e.m.

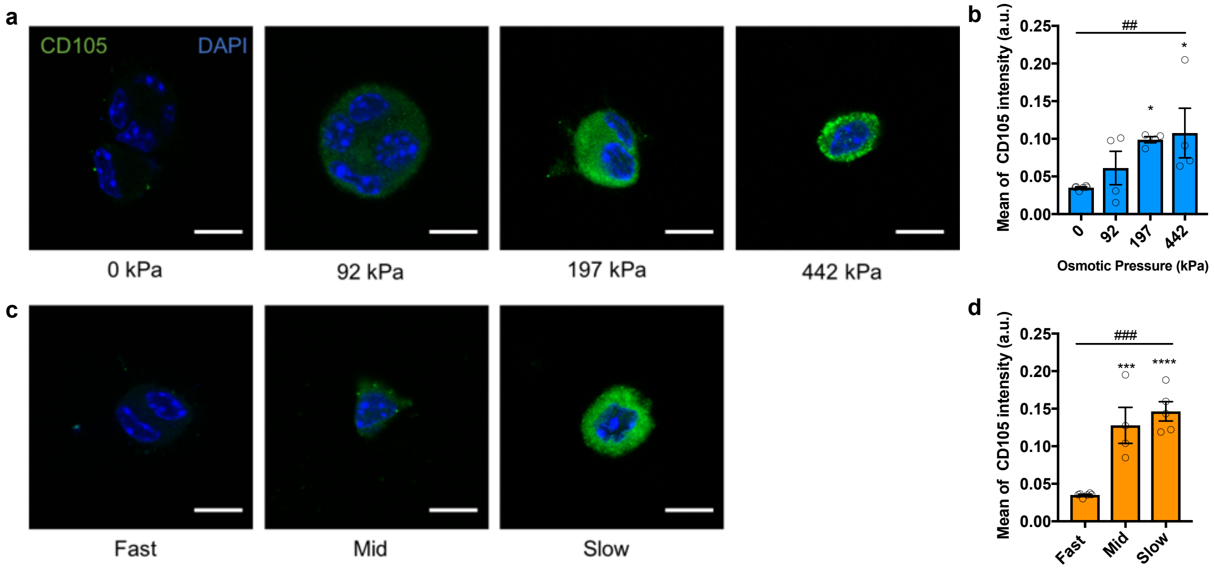

Supplementary Figure 7 | MSCs maintain stemness in conditions in which osteogenic differentiation is diminished. a-d, Representative images of CD105 (green), a marker of stemness, and quantification of CD105 intensity of MSCs cultured in fast relaxing hydrogels with altered osmotic pressure (a, b) and in hydrogels with varying stress relaxation (c, d) for 7 days. ($n \geq 4$ images per each condition, **** $p<0.0001$, *** $p<0.001$, and * $p<0.05$ compared to fast relaxing hydrogel and non-osmotic pressure condition by one-way ANOVA test, ## $p<0.01$ and ### $p<0.001$ by Spearman's rank correlation). All data are shown as mean ± s.e.m.

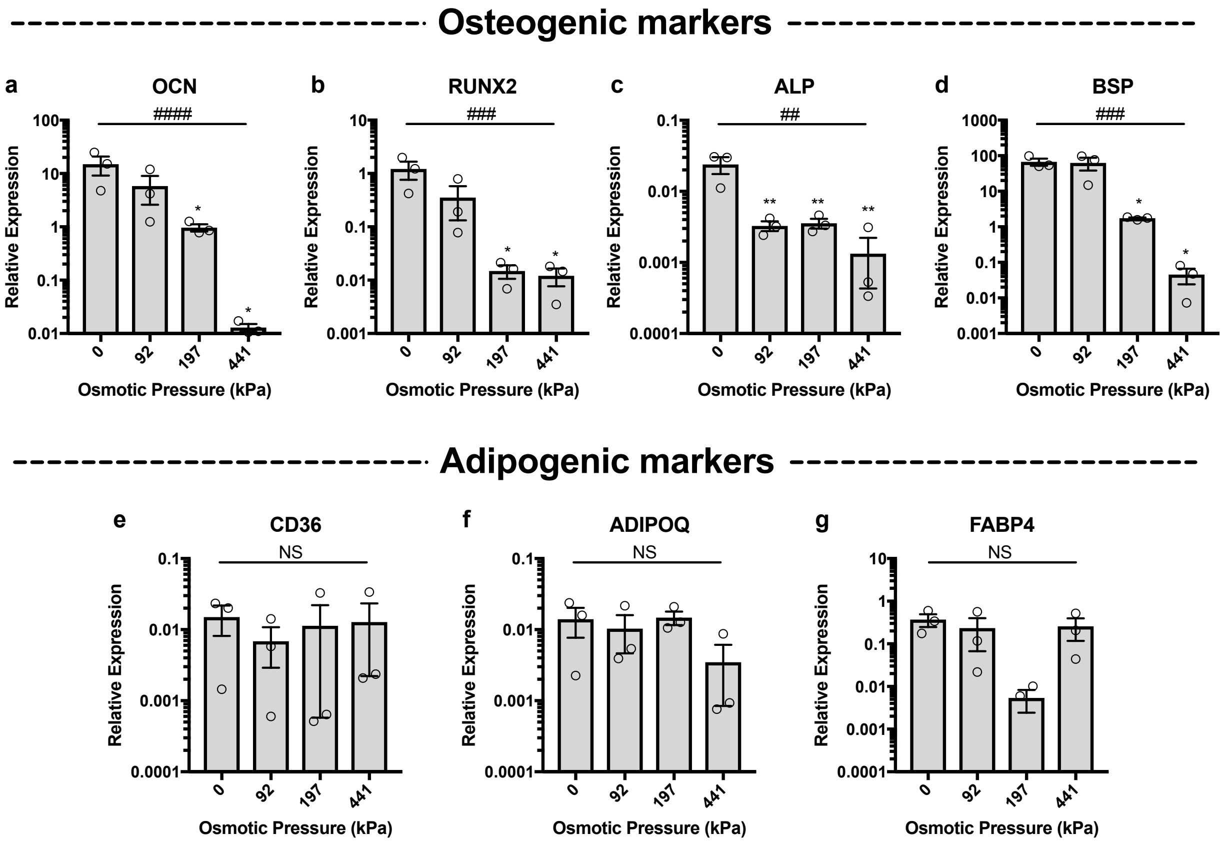

Supplementary Figure 8 | Expression of osteogenic and adipogenic genes in hydrogels with varying levels of hyper-osmotic pressure a-d, Relative mRNA expression of osteogenic markers for MSCs cultured in fast relaxing hydrogels with altered osmotic pressure for 7 days: Osteocalcin (OCN, a), Runt- related transcription factor 2 (RUNX2, b), Alkaline phosphatase (ALP, c), and Bone sialoprotein (BSP, d). **e-g,** Relative mRNA expression of adipogenic markers for MSCs cultured in fast relaxing hydrogels with altered osmotic pressure for 7 days: Cluster of differentiation 36 (CD36, e), Adiponectin (ADIPOQ, f), and Fatty acid binding protein (FAPB4, g). (n = 3 biological replications per each condition, ** $p<0.01$, and * $p<0.05$ compared to non-osmotic pressure condition by one-way ANOVA test, #### $p<0.0001$, ### $p<0.001$, ## $p<0.01$, and NS $p>0.05$ by Spearman's rank correlation). All data are shown as mean ± s.e.m

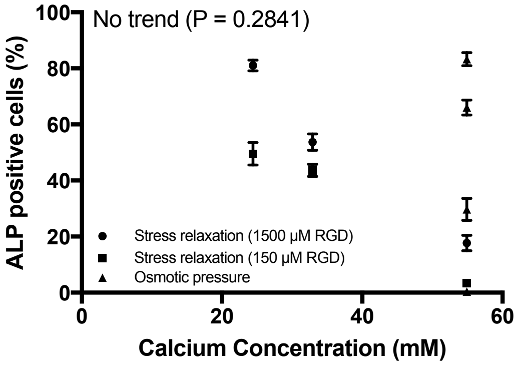

Supplementary Figure 9 | ALP-positive cell fraction is not correlated with calcium crosslinker concentration. Quantification of the percentage of cells positively stained for ALP produced by MSCs cultured in gels in the indicated conditions as a function of the concentration of calcium used to crosslink the matrix. There is no correlation between calcium concentration and the fraction of ALP positive cells (Spearman rank correlation, p = 0.2841, n=3 biological replicates per conditions).

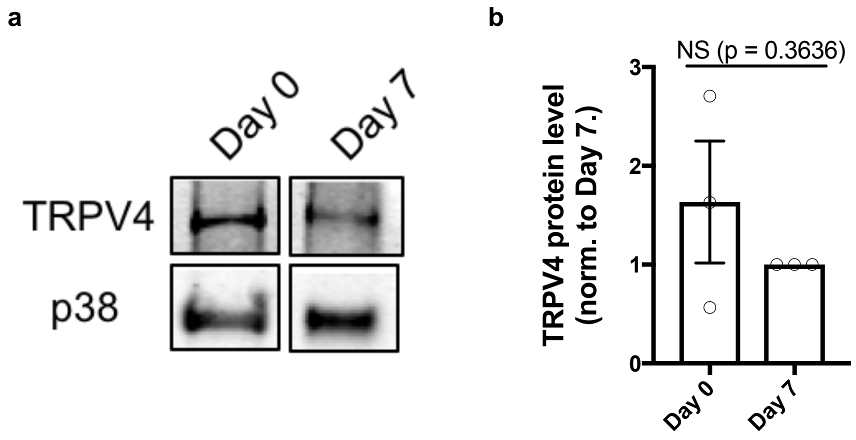

Supplementary Figure 10 | TRPV4 expression over time in hydrogels with fast stress relaxation. a-b, Western blot analysis (a) and quantification (b) of TRPV4 protein expression in MSCs before culturing in hydrogel with fast stress relaxation and after culturing for 7 days (NS, $p = 0.3636$ by student t-test). Data are shown as mean ± s.e.m.

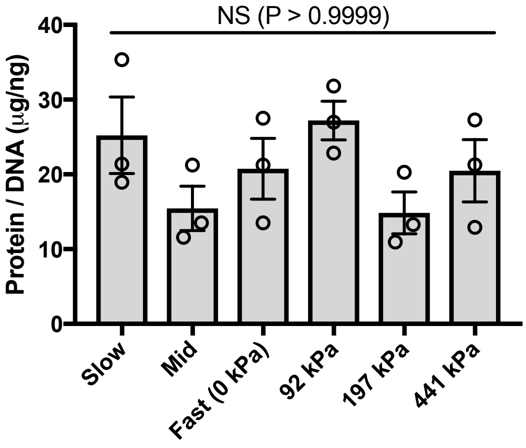

Supplementary Figure 11 | Amount of total protein for MSCs is similar across hydrogels with varying levels of stress relaxation or hyper-osmotic pressure. Quantification of the amount of proteins in cells after 7 days of culture in the indicated conditions, normalized to DNA amounts (n=3 replicates per condition, NS $p>0.9999$ by Spearman's rank correlation).

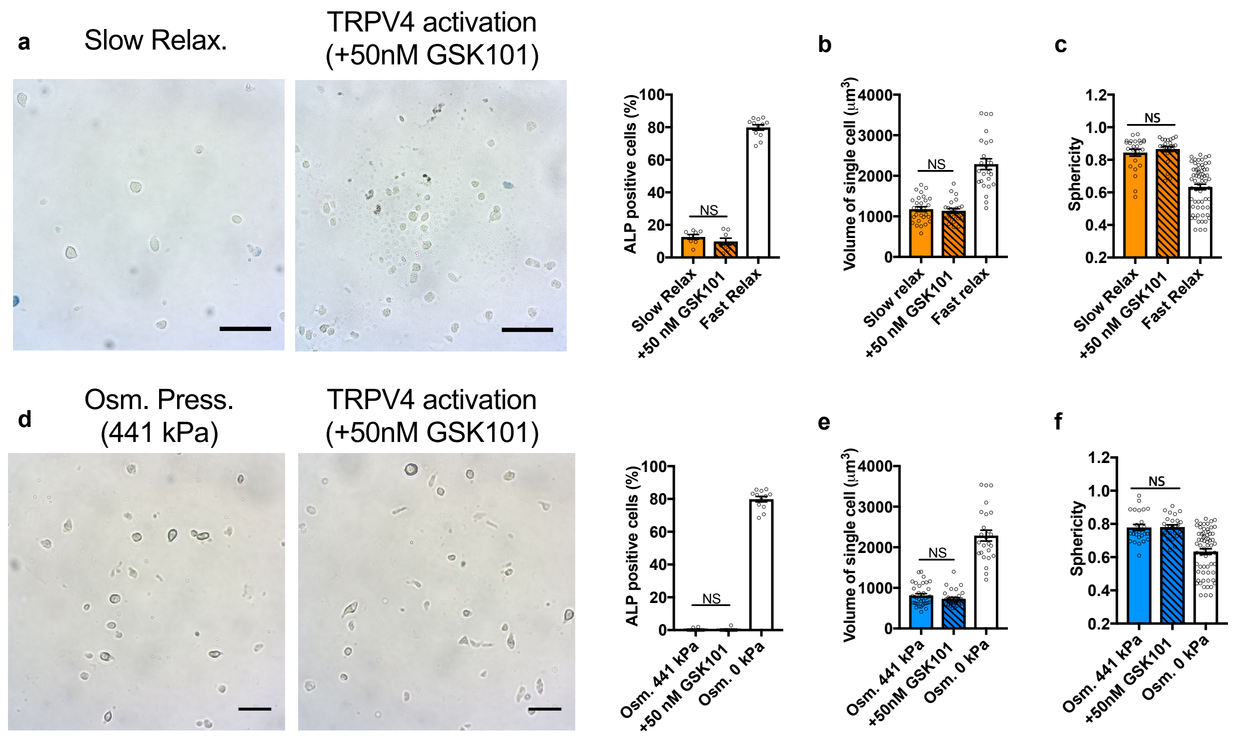

Supplementary Figure 12 | Osteogenic differentiation and volume expansion of MSCs cultured under strongly confining environments treated with the TRPV4 agonist for 7 days. **a**, Representative images of ALP staining and quantifications of the percentage of MSCs positively stained for ALP in slow relaxing hydrogels with or without treatment of the TRPV4 agonist for 7 days (n ≥ 10 images from 3 biological replications per each condition, NS p>0.05 compared to control by one-way ANOVA test). Scale bar, 25 μm **b-c,** Quantification of cell volumes (**b**) and sphericity (**c**) of MSCs cultured in slow relaxing gels with indicated conditions for 7 days (n ≥ 30 single cells from 3 biological replications per each condition, NS indicates p>0.05 by student t-test). **d**, Representative images of ALP staining and quantifications of the percentage of MSCs positively stained for ALP in fast relaxing hydrogel under hyper-osmotic pressure (441 kPa) with or without treatment of the TRPV4 agonist for 7 days (n ≥ 10 images per each condition, NS p>0.05 compared to control by one-way ANOVA test). Scale bar, 25 μm **e-f,** Quantification of cell volumes (**e**) and sphericity (**f**) of MSCs cultured in fast relaxing gels under hyper-osmotic

pressure (441 kPa) with indicated conditions for 7 days ($n \geq 30$ single cells per each condition, NS indicates $p>0.05$ by student t-test). All data are shown as mean ± s.e.m.

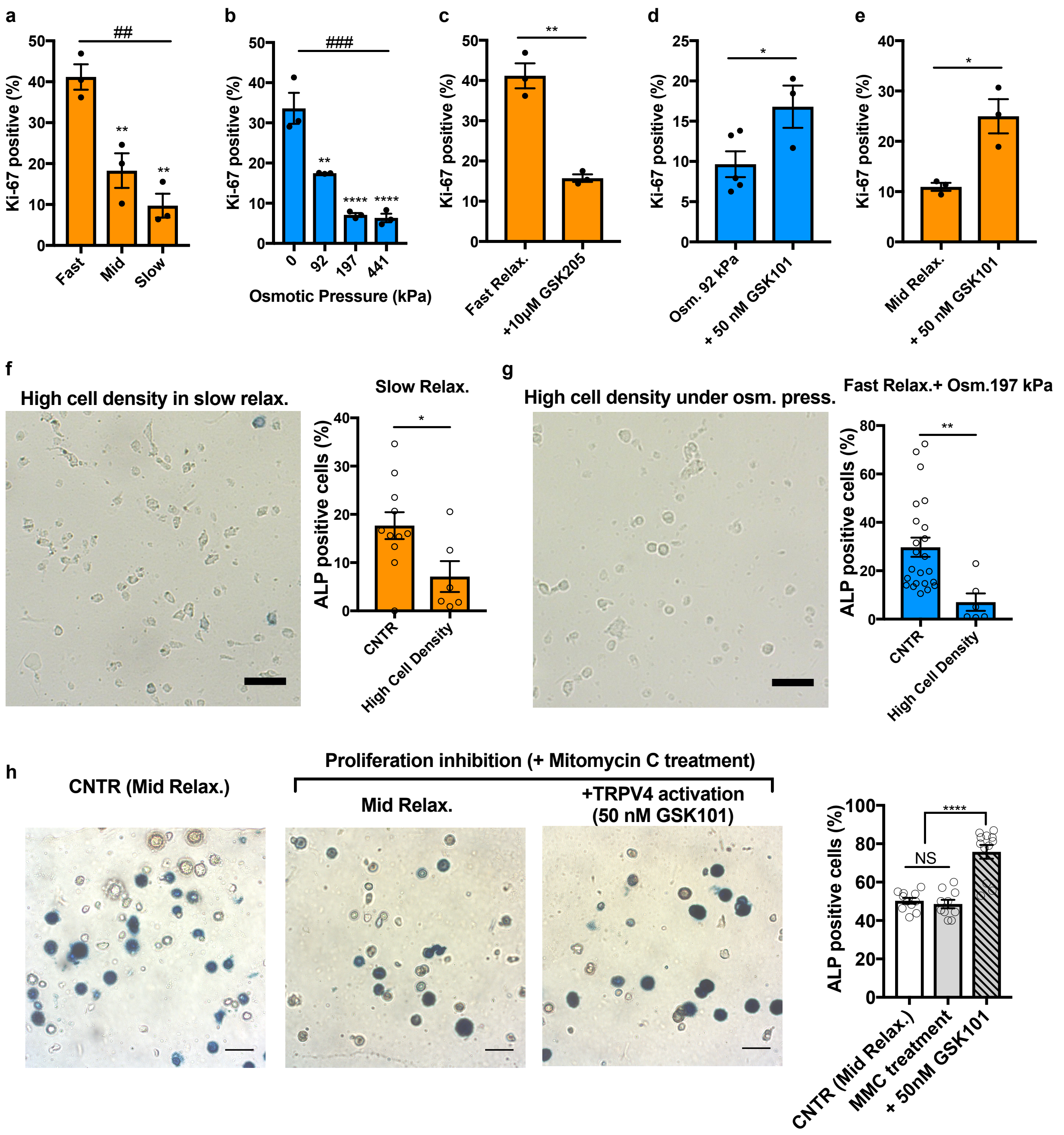

Supplementary Figure 13 | Higher cell density and proliferation does not mediate impact of stress relaxation, or hyper-osmotic pressure, on osteogenic differentiation. a-b, Quantification of Ki-67 staining of MSCs cultured in hydrogels with different stress relaxation (a) and in fast relaxing hydrogels with altered osmotic pressure over 7 days (b). Ki-67 is a marker of cell-cycle progression. **c-e,** Quantifications of Ki-67 staining of MSCs cultured in fast relaxing

hydrogels with TRPV4 inhibition (c) and under osmotic pressure with TRPV4 activation (d) and in mid relaxing hydrogels with TRPV4 activation (e) for 7 days. ($n = 3$ replicates, measured in 100 cells per replicate, **** $p<0.0001$, ***$p<0.001$, **$p<0.01$, and *$p<0.05$ by one-way ANOVA test in a-b and student t-test in c-e, ## and ### on the top in figure a-b indicate $p<0.01$ and $p<0.001$ by Spearman's rank correlation, respectively). **f-g,** Representative images of ALP staining and quantifications of the percentage of MSCs positively stained for ALP in slow relaxing hydrogels without osmotic pressure (f) and in fast relaxing hydrogel with osmotic pressure (g), both cultured at higher cell densities for 7 days. Scale bar, 25 µm ($n \geq 5$ images from 3 replications per each condition, * $p<0.05$ and ** $p<0.01$ compared to control by student t-test). **h,** Representative images of ALP staining and quantifications of the percentage of MSCs positively stained for ALP in mid relaxing hydrogels. MSCs were treated with mitomycin-C before encapsulation and cultured with or without treatment of the TRPV4 agonist for 7 days. Scale bar, 25 µm ($n \geq 5$ images from 3 replications per each condition, **** $p<0.0001$ by student t-test). All data are shown as mean ± s.e.m.

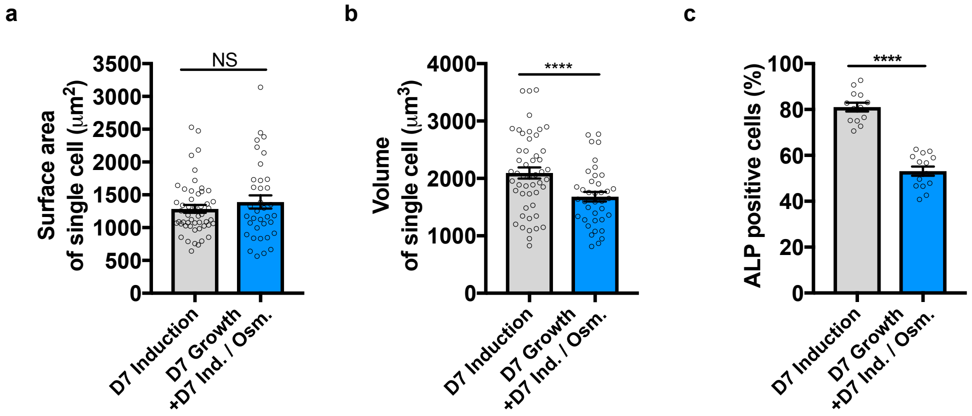

Supplementary Figure 14 | Surface area of single cell is not correlated to osteogenic differentiation. a-b, Quantification of cell surface area (a) and volume (b) of single cells cultured in indicated conditions (n ≥ 35 single cells from 3 biological replications per each condition, **** p<0.0001and NS p>0.05 by student t-test). **c,** Quantification of the percentage of cells positively stained for alkaline phosphatase produced by MSCs cultured in indicated conditions (n ≥ 12 images from 3 biological replications per each condition, **** p<0.0001 by student t-test). Data are shown as mean ± s.e.m.

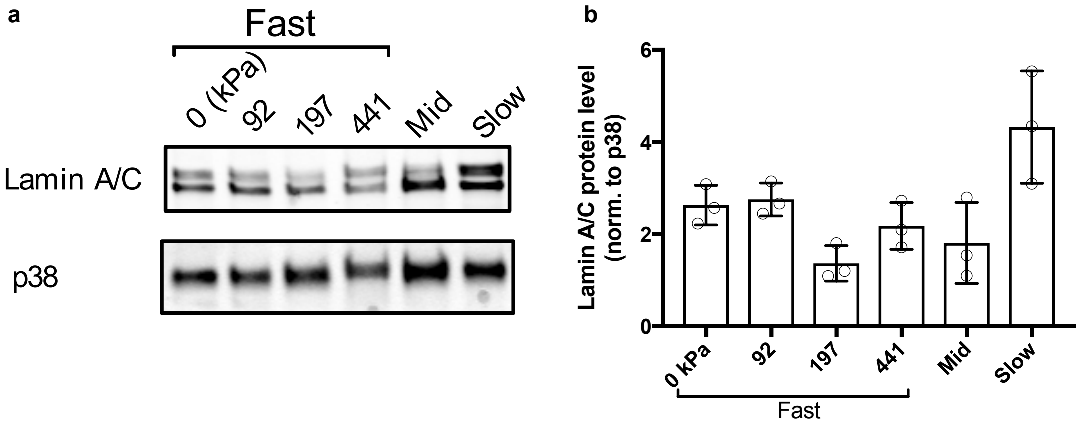

Supplementary Figure 15 | Nuclear lamin levels are similar for MSCs in hydrogels with varying levels of stress relaxation or hyperosmotic pressure. a-b, Western blot analysis (a) and quantification (b) of lamin protein levels in MSCs cultured in hydrogel with varying stress relaxation and in fast relaxing hydrogel with altered osmotic pressure for 7 days (** $p<0.01$, and * $p<0.05$ respectively compared to fast stress relaxation and 0 kPa osmotic pressure case by one-way ANOVA test).

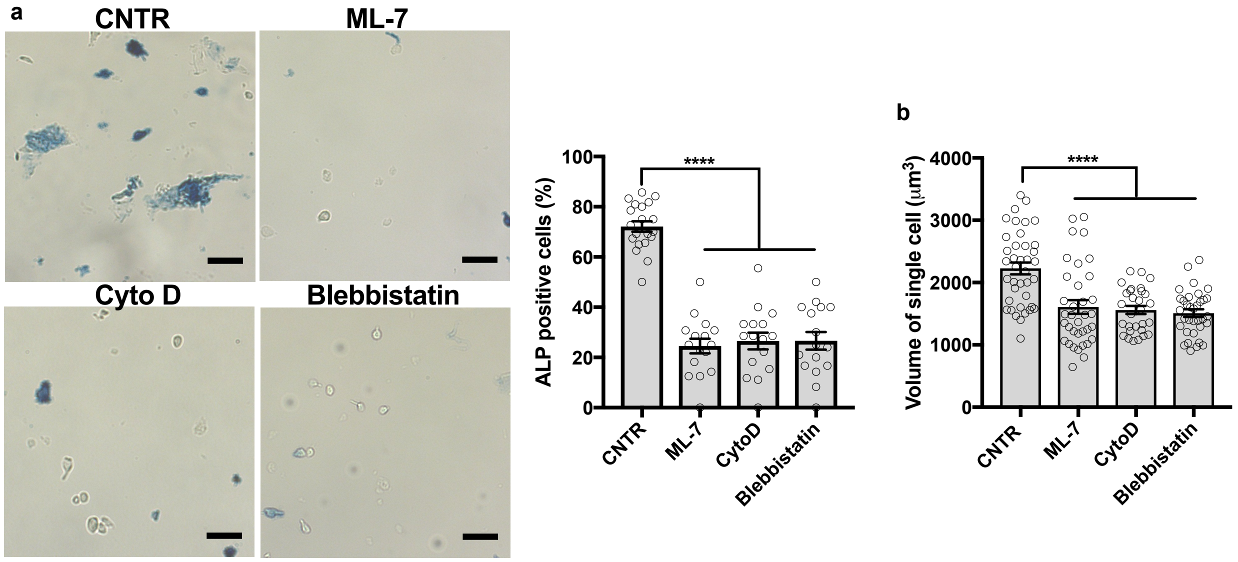

Supplementary Figure 16 | Pharmacological inhibition of MLCK with ML-7, actin polymerization with cytochalasin D, and myosin with blebbistatin diminishes cell volume expansion and osteogenic differentiation. a, Representative images of ALP staining and quantifications of the percentage of MSCs positively stained for ALP in fast relaxing hydrogels with indicated inhibitors for 7 days ($n \geq 15$ images from 3 replications per each condition). Scale bar, 25 μm. **b,** Quantification of volume of single cells cultured in indicated conditions ($n \geq 35$ single cells from 3 replications per each condition). **** indicates $p<0.0001$ compared to control by one-way ANOVA test. All data are shown as mean ± s.e.m.

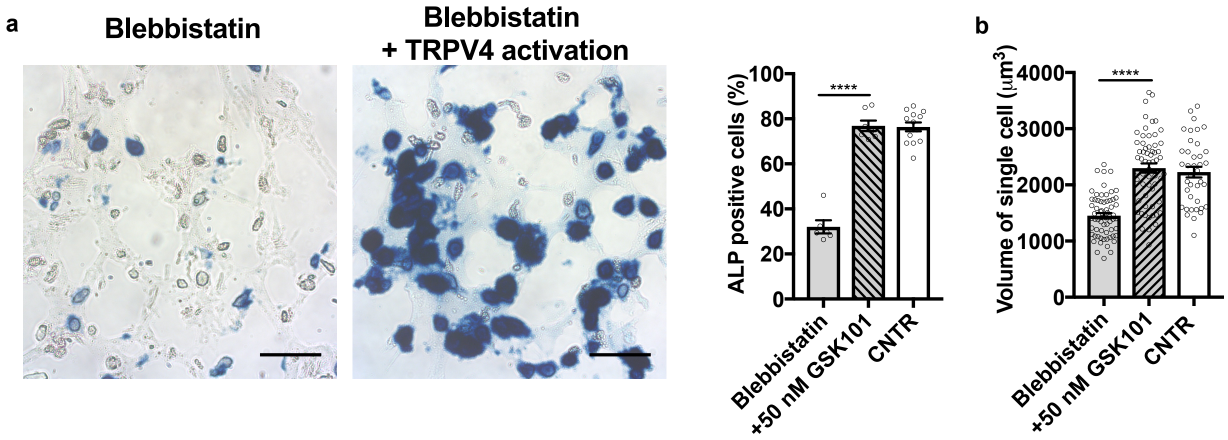

Supplementary Figure 17 | TRPV4 activation restores the osteogenic differentiation diminished by pharmacological inhibition of myosin with blebbistatin in fast relaxing hydrogels. a, Representative images of ALP staining and quantifications of the percentage of MSCs positively stained for ALP in fast relaxing hydrogels treated with blebbistatin and with or without treatment of the TRPV4 agonist for 7 days (n ≥ 6 images from 3 replications per each condition). Scale bar, 25 μm. **b,** Quantification of volume of single cells cultured in indicated conditions (n ≥ 35 single cells from 3 replications per each condition). **** indicates p<0.0001 compared to control by one-way ANOVA test. All data are shown as mean ± s.e.m.

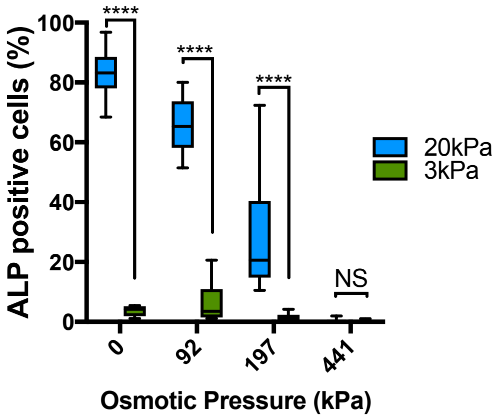

Supplementary Figure 18 | Osteogenesis of MSCs is dependent on hydrogel stiffness and hyper-osmotic pressure. Quantification of the percentage of cells positively stained for alkaline phosphatase produced by MSCs cultured in gels with initial modulus 3kPa and 20 kPa under the indicated osmotic pressure ($n \geq 10$ images from 3 replications per each condition, **** $p<0.0001$ and NS $p>0.5$ by two-way ANOVA). The box plots show 25/50/75th percentiles and whiskers show minimum/maximum. All data are shown as mean ± s.e.m.

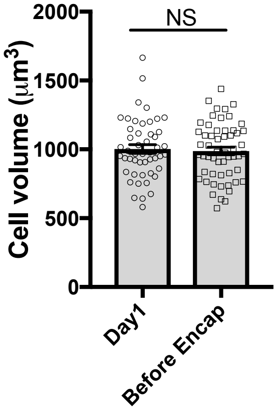

Supplementary Figure 19 | Cell volume before and after encapsulation. Quantification of volume of single cells before encapsulation and cultured in hydrogels for 1 day ($n \geq 48$ single cells from 3 replicates per each condition). NS indicates $p = 0.7245$ by student t-test. All data are shown as mean ± s.e.m.

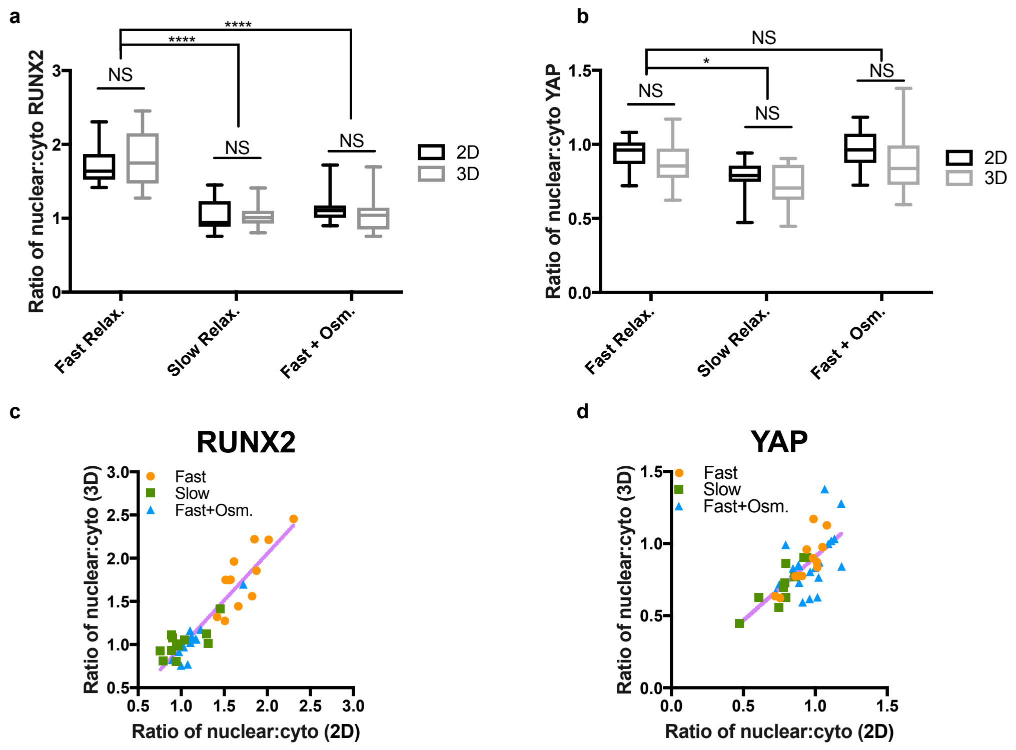

Supplementary Figure 20 | Comparison of YAP and RUNX2 localization measurements of 2D images and full 3D stacks. a-b, Quantification of the ratio of nuclear protein (a, RUNX2 or b, YAP) to the protein (a, RUNX2 or b, YAP) in the cytoplasm (**** $p<0.0001$ * $p<0.05$, and NS $p>0.8$ by two-way ANOVA test). The box plots show 25/50/75th percentiles and whiskers show minimum/maximum. All data are shown as mean ± s.e.m. **c-d,** Linear regression analysis indicated a linear correlation between two ratios of nuclear localization of RUNX2 (c) and YAP (d) with 2D images and 3D stack images (R square = 0.8532 for RUNX2 and R square = 0.5037 for YAP, n ≥ 40 single cells from 3 biological replications per each condition).

Gene	Forward Sequence	Reverse Sequence
OCN	CCGGGAGCAGTGTGAGCTTA	AGGCGGTCTTCAAGCCATACT
RUNX2	AGGGACTATGGCGTCAAACA	GGCTCACGTCGCTCATCTT
ALP	GGACAGGACACACACACACA	CAAACAGGAGAGCCACTTCA
BSP	ACAATCCGTGCCACTCACT	TTTCATCGAGAAAGCACAGG
CD36	TCCTCTGACATTTGCAGGTCTATC	AAAGGCATTGGCTGGAAGAA
ADIPOQ	TGFTCCTAATCCTGCCCA	CCAACCTGCACAAGTTCCCTT
FABP4	CGACAGGAAGGTGAAGAGCA	ATTCCACCACCAGCTTGTCA
GAPDH	TGCACCACCAACTGCTTAG	GGATGCAGGGATGATGTTC

Supplementary Table 1 | List of specific primers for real-time polymerase chain reaction

Figure	Analysis	One- tail or Two-tail	F (dfn, dfd) or T, df
Figure 1b	One-way ANOVA	Two-tail	F (3, 146) = 53.38
Figure 1c	One-way ANOVA	Two-tail	F (3, 167) = 33.53
Figure 1f	Student t-test	Two-tail	t=3.049 df=16
Figure 1g	Two-way ANOVA	Two-tail	F (2, 212) = 53.64
Figure 1h	Two-way ANOVA	Two-tail	F (2, 243) = 25.74
Figure 2b	One-way ANOVA	Two-tail	F (4, 184) = 57.54
Figure 2c	One-way ANOVA	Two-tail	F (4, 181) = 39.51
Figure 2e	Two-way ANOVA	Two-tail	F (3, 54) = 91.77
Figure 2h	One-way ANOVA	Two-tail	F (3, 119) = 23.97
Figure 2i	One-way ANOVA	Two-tail	F (3, 118) = 6.651
Figure 2k	Two-way ANOVA	Two-tail	Time: F (2, 43) = 328.8<br>Osm: F (1, 43) = 17.04
Figure 3a	Two-way ANOVA	Two-tail	Relax: F (2, 8) = 8.416<br>Osm.: F (3, 8) = 11.5
Figure 3c	One-way ANOVA	Two-tail	F (2, 39) = 20.88
Figure 3d	One-way ANOVA	Two-tail	F (3, 52) = 20.01
Figure 4b	One-way ANOVA	Two-tail	F (2, 27) = 112.1
Figure 4c	One-way ANOVA	Two-tail	F (2, 105) = 64.41
Figure 4d	One-way ANOVA	Two-tail	F (2, 84) = 27.67
Figure 4e	Student t-test	Two-tail	t=5.567 df=22
Figure 4f	Student t-test	Two-tail	t=4.311 df=19
Figure 4g	Student t-test	Two-tail	t=3.748 df=63
Figure 4h	Student t-test	Two-tail	t=5.619 df=64
Figure 5a	Student t-test	Two-tail	t=0.08987 df=63
Figure 5b	Student t-test	Two-tail	t=0.9227 df=51
Figure 5f	One-way ANOVA	Two-tail	F (2, 112) = 17.72
Figure 5g	One-way ANOVA	Two-tail	F (2, 120) = 29.27
Figure 5i	Student t-test	Two-tail	t=8.551 df=24
Figure 6a	One-way ANOVA	Two-tail	F (2, 177) = 24.84
Figure 6b	One-way ANOVA	Two-tail	F (3, 244) = 0.01044
Figure 6c	One-way ANOVA	Two-tail	F (2, 140) = 32.28
Figure 6d	One-way ANOVA	Two-tail	F (3, 230) = 35.52
Figure 6e	Two-way ANOVA	Two-tail	Time: F (2, 43) = 328.8<br>Osm: F (1, 43) = 17.04
Figure 6f	Student t-test	Two-tail	t=9.824 df=71
Figure 6g	Student t-test	Two-tail	t=5.212 df=70
Figure 6i	Student t-test	Two-tail	t=13.44 df=34
Figure 6j	Student t-test	Two-tail	t=7.108 df=57
Figure 6k	Student t-test	Two-tail	t=0.1391 df=69
Supplementary Figure 1b	One-way ANOVA	Two-tail	F (2, 9) = 15.25
Supplementary Figure 1c	One-way ANOVA	Two-tail	F (2, 9) = 0.5413
Supplementary Figure 2a	One-way ANOVA	Two-tail	F (3, 133) = 40.05
Supplementary Figure 2b	One-way ANOVA	Two-tail	F (3, 143) = 34.86
Supplementary Figure 2e	One-way ANOVA	Two-tail	F (2, 32) = 0.1649
Supplementary Figure 2f	One-way ANOVA	Two-tail	F (2, 433) = 18.67
Supplementary Figure 3b	One-way ANOVA	Two-tail	F (3, 160) = 28.94
Supplementary Figure 3c	One-way ANOVA	Two-tail	F (3, 156) = 15.53
Supplementary Figure 3e	One-way ANOVA	Two-tail	F (2, 30) = 75.53
Supplementary Figure 4b	One-way ANOVA	Two-tail	F (5, 12) = 5.391
Supplementary Figure 5	One-way ANOVA	Two-tail	F (4, 178) = 81.34
Supplementary Figure 6	One-way ANOVA	Two-tail	F (2, 36) = 27.09
Supplementary Figure 7b	One-way ANOVA	Two-tail	F (3, 14) = 4.039

Supplementary Figure 7d	One-way ANOVA	Two-tail	F (2, 12) = 22.85
Supplementary Figure 8a	One-way ANOVA	Two-tail	F (3, 8) = 4.264
Supplementary Figure 8b	One-way ANOVA	Two-tail	F (3, 8) = 5.054
Supplementary Figure 8c	One-way ANOVA	Two-tail	F (3, 8) = 10.67
Supplementary Figure 8d	One-way ANOVA	Two-tail	F (3, 8) = 6.513
Supplementary Figure 8e	One-way ANOVA	Two-tail	F (3, 8) = 0.1625
Supplementary Figure 8f	One-way ANOVA	Two-tail	F (3, 8) = 1.195
Supplementary Figure 8g	One-way ANOVA	Two-tail	F (3, 8) = 1.5
Supplementary Figure 9	Spearman's correl.	Two-tail	r = -0.4964
Supplementary Figure 10b	Student t-test	Two-tail	t=1.024 df=4
Supplementary Figure 11	One-way ANOVA	Two-tail	F (5, 12) = 1.797
Supplementary Figure 12a	Student t-test	Two-tail	t=1.104 df=14
Supplementary Figure 12b	Student t-test	Two-tail	t=0.483 df=49
Supplementary Figure 12c	Student t-test	Two-tail	t=0.8218 df=46
Supplementary Figure 12d	Student t-test	Two-tail	t=0.1675 df=18
Supplementary Figure 12e	Student t-test	Two-tail	t=1.547 df=70
Supplementary Figure 12f	Student t-test	Two-tail	t=0.1236 df=49
Supplementary Figure 13a	One-way ANOVA	Two-tail	F (2, 6) = 21.93
Supplementary Figure 13b	One-way ANOVA	Two-tail	F (3, 8) = 39.73
Supplementary Figure 13c	Student t-test	Two-tail	t=7.851 df=4
Supplementary Figure 13d	Student t-test	Two-tail	t=2.487 df=6
Supplementary Figure 13e	Student t-test	Two-tail	t=4.015 df=4
Supplementary Figure 13f	Student t-test	Two-tail	t=2.365 df=15
Supplementary Figure 13g	Student t-test	Two-tail	t=2.817 df=27
Supplementary Figure 13h	One-way ANOVA	Two-tail	F (2, 30) = 33.83
Supplementary Figure 14a	Student t-test	Two-tail	t=0.9569 df=84
Supplementary Figure 14b	Student t-test	Two-tail	t=4.766 df=98
Supplementary Figure 14c	Student t-test	Two-tail	t=10.1 df=25
Supplementary Figure 15b	One-way ANOVA	Two-tail	F (5, 12) = 6.369
Supplementary Figure 16a	One-way ANOVA	Two-tail	F (3, 64) = 69.19
Supplementary Figure 16b	One-way ANOVA	Two-tail	F (3, 133) = 15.65
Supplementary Figure 17a	One-way ANOVA	Two-tail	F (2, 23) = 95.4
Supplementary Figure 17b	One-way ANOVA	Two-tail	F (2, 161) = 46.26
Supplementary Figure 18	Two-way ANOVA	Two-tail	F (1, 74) = 213.7
Supplementary Figure 19	Student t-test	Two-tail	t=0.3534 df=102
Supplementary Figure 20a	Two-way ANOVA	Two-tail	Exp. Cond.: F (2, 66) = 63.39<br>Dim.: F (1, 66) = 0.007808
Supplementary Figure 20b	Two-way ANOVA	Two-tail	Exp. Cond.: F (2, 84) = 9.719<br>Dim.: F (1, 84) = 4.768

Supplementary Table 2 | Details for statistical analyses T indicates t-values for t-tests, F indicates F-values for ANOVA tests, and df indicates degrees of freedom.
